# *CGB *and *GNRH1 *expression analysis as a method of tumor cells metastatic spread detection in patients with gynecological malignances

**DOI:** 10.1186/1479-5876-9-130

**Published:** 2011-08-09

**Authors:** Mirosław Andrusiewicz, Anna Szczerba, Maria Wołuń-Cholewa, Wojciech Warchoł, Ewa Nowak-Markwitz, Emilia Gąsiorowska, Krystyna Adamska, Anna Jankowska

**Affiliations:** 1 Department of Cell Biology, Poznan University of Medical Sciences, Rokietnicka Street 5D, 60-806 Poznan, Poland; 2 Department of Biophysics, Poznan University of Medical Sciences, Fredry Street 10, 61-701 Poznan, Poland; 3 Department of Gynecologic Oncology, Poznan University of Medical Sciences, Polna Street 33, 60-535 Poznan, Poland; 4 The Great Poland Cancer Center in Poznan, Garbary Street 15, 61-688 Poznan, Poland

**Keywords:** human chorionic gonadotropin beta subunit, gonadotropin releasing hormone type 1, real time RT-PCR, CTC

## Abstract

**Background:**

Metastasis is a common feature of many advanced stage cancers and metastatic spread is thought to be responsible for cancer progression. Most cancer cells are localized in the primary tumor and only a small population of circulating tumor cells (CTC) has metastatic potential. CTC amount reflects the aggressiveness of tumors, therefore their detection can be used to determine the prognosis and treatment of cancer patients.

The aim of this study was to evaluate human chorionic gonadotropin beta subunit (CGB) and gonadoliberin type 1 (GNRH1) expression as markers of tumor cells circulating in peripheral blood of gynecological cancer patients, indicating the metastatic spread of tumor.

**Methods:**

*CGB *and *GNRH1 *expression level in tumor tissue and blood of cancer patients was assessed by real-time RT-PCR. The data was analyzed using the Mann-Whitney U and Spearman tests. In order to distinguish populations with homogeneous genes' expression the maximal likelihood method for one- and multiplied normal distribution was used.

**Result:**

Real time RT-PCR results revealed *CGB *and *GNRH1 *genes activity in both tumor tissue and blood of gynecological cancers patients. While the expression of both genes characterized all examined tumor tissues, in case of blood analysis, the transcripts of *GNRH1 *were found in all cancer patients while *CGB *were present in 93% of patients. *CGB *and *GNRH1 *activity was detected also in control group, which consisted of tissue lacking cancerous changes and blood of healthy volunteers. The log-transformation of raw data fitted to multiplied normal distribution model showed that *CGB *and *GNRH1 *expression is heterogeneous and more than one population can be distinguished within defined groups.

Based on *CGB *gene activity a critical value indicating the presence of cancer cells in studied blood was distinguished. In case of *GNRH1 *this value was not established since the results of the gene expression in blood of cancer patients and healthy volunteers were overlapping. However one subpopulation consists of cancer patient with much higher *GNRH1 *expression than in control group was found.

**Conclusions:**

Assessment of *CGB *and *GNRH1 *expression level in cancer patients' blood may be useful for indicating metastatic spread of tumor cells.

## Background

Neoplastic diseases represent chaotic self-developing systems, in which genetically destabilized cells replicate themselves continuously [1]. Within each replication cycle they produce new, modified daughter cells [2,3]. The accumulation of genetic alternations increases genetic instability [4]. During this process several different cell lines with different gene expression profile might co-exist within one tumor [5-10]. Cancer cells and their metastatic progeny retain the capacity for self-evolution [1]. New cell variants are better adapted to local growth requirements and might survive or undergo apoptosis [11,12].

Tumors with a high degree of genetic instability are able to produce more cells, thereby providing a larger reservoir for new, better adapted variants. This corresponds to development from preneoplastic to invasive cancer and consequently worse prognosis [4,13-15].

Some cancer cells posses the ability to penetrate the walls of blood vessels, circulate in the bloodstream and reach other niches of the body. These circulating tumor cells (CTC) are thought to be responsible for metastatic spread and cancer progression. Therefore detection of circulating tumor cells may be important for both diagnosis and treatment of cancer patients [16-19].

While most cancer cells (CC) are localized in the primary tumor, there is only a small population of circulating cancer cells having metastatic potential. The frequency of CTC occurrence in peripheral blood is estimated to be 1 cancer cell per 10^5-7 ^mononuclear cells [20]. Nevertheless their presence and amount reflect the aggressiveness of tumors [21,22].

Recently highly sensitive methods have been developed to detect CTC in blood of cancer patients. These methods include flow cytometry, immunohistochemistry and real time RT-PCR [23-27]. Still, most of these methods do not seem to be sensitive enough to detect CTC in patients with early-stage carcinomas [28-31].

The objective of this study was to use quantitative real time RT-PCR and analyze the expression level of two genes: human chorionic gonadotropin beta subunit (*CGB*) and gonadotropin releasing hormone type 1 (gonadoliberin type 1, *GNRH1*) in order to detect CTC in peripheral blood of gynecological cancer patients. The research was undertaken to establish the sensitivity and specificity of the genes activity as an informative way to identify tumor cells of gynecological origin in blood of cancer patients, which can indicate metastatic spread of tumor cells.

These two genes were selected because a number of studies have demonstrated that their expression level is up-regulated in gynecological tumors [32-38].

Serum free CGB or its urinary degradation product beta-core fragments are found in 68% of ovarian, 51% of endometrial and 46% of cervical malignancies [32]. Our earlier study proved that CGB is expressed by analyzed gynecological tumor tissues [33-35]. The free beta subunit of human chorionic gonadotropin was originally considered as biologically non-functional, however it was shown recently that CGB may stimulate tumor growth and inhibit its apoptosis. This theory is supported by the results of *CGB *genes silencing, showing that reduction of the hormone's expression *in vitro *resulted in increased apoptosis rate of cancer cells [36]. Furthermore elevated CGB level in serum was found to be associated with higher aggressiveness of cancer and its resistance to therapy [32].

In ovarian, endometrial, mammary, and prostate cancers significant level of GNRH1 expression was also detected and the agonists of GNRH1 have been shown to inhibit proliferation and stimulate apoptosis of ovarian and endometrial carcinoma cells [37]. We have previously demonstrated that the expression of *CGB *in endometrial cancer as well as in endometrial atypical hyperplasia is accompanied by expression of gonadotopin releasing-hormone type 1 [38].

In this study we showed that the up-regulation of human chorionic gonadotropin beta subunit and gonadoliberin type 1 genes expression may indicate the presence of tumor cells circulating in peripheral blood of gynecological cancer patients. Thus, the expression of *CGB *and *GNRH1 *may become a prognostic factor of metastatic spread of tumor cells [38].

## Materials and methods

### Patients

Surgical specimens of gynecological cancer tissue have been obtained from 48 patients (age range 36-79) treated with surgery at the Department of Gynecologic Oncology, Poznan University of Medical Sciences. Peripheral blood from 41 cancer patients (age range 36-79) was collected before surgery. None of the patients received chemo- or radiotherapy prior to the operation. Histology groups were as follows: ovarian carcinoma (25 cases; FIGO: I, n = 4; II, n = 1; III, n = 14; not determinate, n = 6), endometrial carcinoma (14 cases, FIGO not evaluated), uterine cervix carcinoma (9 cases; FIGO 0, n = 1; I, n = 4; II, n = 2; III, n = 0; not determinate, n = 2).

The control group consisted of blood from 43 healthy volunteers (age range 21 - 56) and 12 control tissue samples lacking pathological changes. The absence of cancerous changes has been confirmed by anatomicopathologic macroscopic and microscopic examinations. These tissue samples were obtained from patients operated for reasons other than cancer. The study was approved by the Institutional Ethics Review Board of Poznan University of Medical Sciences. All patients and volunteers participated in the research after obtaining informed consent.

### Sample collection

9 ml of blood from the patients and from the volunteers was collected in S-monovette tubes (SARSTEDT AG & Co., Numbrecht, Germany). The blood samples where diluted with PBS (without Ca^2+ ^and Mg^2+^) up to 17 ml. The PfU blood separation tubes and LSM 1077 separation medium (PAA Laboratories GmbH, Pasching, Austria) were used to separate the cells during centrifugation at 1200 × g for 20 minutes at room temperature in a swinging bucket rotor. Cells located in the interphase were collected and washed twice with 10 ml of PBS. The cells were resuspended in 1.5 ml TRIzol LS Reagent (Invitrogen, CA, USA) and stored at -80°C until total RNA isolation was performed.

Tissue samples from patients after surgical removal were placed in RNA*Later *and stored at -80°C.

### RNA isolation and cDNA synthesis

Total cellular RNA from blood and tissue samples was extracted with TRIzol LS Reagent (Invitrogen) and Tri*Pure *Isolation Reagent (Roche Diagnostic GmbH, Mannheim, Germany) respectively, according to manufacturer's protocols. RNA purity and concentration was determined spectrophotometrically and electrophoretically in 1.2% agarose gel containing 1.5% formaldehyde (Sigma-Aldrich, USA) in FA buffer (20 mM MOPS, 5 mM sodium acetate, 1 mM EDTA, 200 mM paraformaldehyde; pH 7.0; Sigma-Aldrich).

2 μg of total RNA was used for cDNA synthesis. Mixture of RNA, universal oligo(d)T_10 _primer and RNase-free water was incubated at 65°C for 10 minutes in order to denature RNA secondary structure. Then the mixture was placed on ice and other components: 500 mM dNTPs, 10 nM DTT, 20 U ribonuclease inhibitor, 5 × reverse transcriptase buffer and 50 U of Transcriptor Reverse Transcriptase were added. mRNA was reversely transcribed at 55°C for 30 minutes. It was followed by enzyme inactivation at 85°C for 5 minutes. cDNA was placed on ice or stored at -20°C until real time PCR was performed. All compounds used for cDNA synthesis were purchased from Roche Diagnostic (Roche Diagnostic, Mannheim, Germany).

### Real time PCR

To asses the expression level of *CGB *[NCBI: NM_000737] and *GNRH1 *[NCBI: NM_000825.3] genes real time PCR with sequence specific primers and LightCycler^® ^TaqMan^® ^Master Kit (Roche Diagnostics) has been performed. PCR reaction mixture contained: 5 μl of cDNA, 1x TaqMan Master mix, 0.1 μM hydrolysis probe (TaqMan) and 0.5 μM of the primers. The primers were designed to be complementary to the splice junction, what excluded the possibility of DNA amplification. Hydrolysis probes and primers used are described in table 1. TaqMan hydrolysis probe for examined genes and phosphoribosyltransferase (*HPRT*) housekeeping gene were purchased from Universal Probe Library (Roche Diagnostic).

**Table 1:** Primers and hydrolysis probes used in real-time PCR.

Gene	TaqMan probe No	Forward primer 5'→3'	Reverse primer 5'→3'
*CGB*	#71 Roche Diagnostic, Cat. No: 04688945001	TACTGCCCCACCATGACC	CACGGCGTAGGAGACCAC

*GNRH1*	#29 Roche Diagnostic, Cat. No: 04687612001	GACCTGAAAGGAGCTCTGGA	CTTCTGGCCCAATGGATTTA

*HPRT*	Human *HPRT *Gene Assay (Roche Diagnostic, Cat. No: 05046157001)		

The program of PCR consisted of 1 cycle of 95°C with a 10 minute hold, followed by 45 cycles of 95°C with a 10 seconds hold, annealing/amplification temperature at 60°C with a 30 seconds hold, and 72°C with a 1 seconds hold for fluorescence data acquisition.

All experiments were performed in triplicates. PCR efficiencies were calculated from the standard curves (SC) generated using serial decimal dilutions of cDNA synthesized from placenta. A relative expression level of analyzed genes was normalized with control gene - *HPRT*. The final step of the expression level analysis was the calculation of the *CGB*/*HPRT *and *GNRH1*/*HPRT *concentration ratio (Cr).

The PCR products were sequenced to confirm their identity.

### Data collection and Statistical analysis

Real time PCR data was assembled using the LightCycler computer application software 4.05 dedicated for the LightCycler 2.0. All data was analyzed using the Statistica Software ver. 6.0 (StatSoft, Poland).

The Mann-Whitney U test was performed and the differences were considered to be statistically significant if *P*-value was lower than 0.05.

*CGB *and *GNRH1 *concentration ratios were log-transformed to achieve normal distribution of data.

In order to distinguish populations with homogeneous genes' expression the maximal likelihood method for one- and multiplied normal distribution was used.

Relative levels of *CGB *and *GNRH1 *expression between studied groups were correlated using Spearman's Rank Correlation test and the results were considered to be statistically significant if *P*-value was lower than 0.05.

## Results

The expression of *CGB *and *GNRH1 *was evaluated for gynecological tumor tissue and peripheral blood of patients with gynecological cancer using real time RT-PCR method. PCR products identity was confirmed by sequencing.

**Figure 1:**
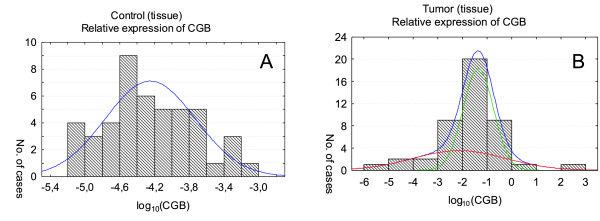
***CGB *gene expression in tissue lacking of cancerous changes (A) and tumor tissue (B)**. Relative expression levels are presented as the logarithm to the base 10. In order to distinguish populations with homogeneous genes' expression the maximal likelihood method for one- and multiplied normal distribution was used. The histograms include one (A) and two (B) normal distribution of *CGB *expression. In case of tumor tissue (B) two normal distributions' sum create the final approximation - higher curve in the graph.

**Figure 2:**
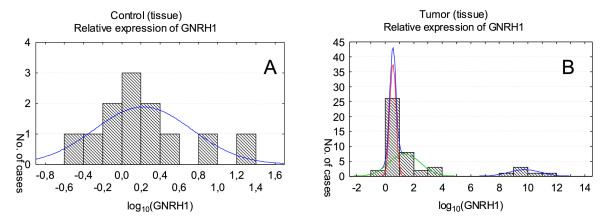
***GNRH1 *expression in tissue lacking cancerous changes (A) and tumor tissue (B)**. Relative expression levels are presented as the logarithm to the base 10. The maximal likelihood method for one- and multiplied normal distribution of *GNRH1 *expression was used and one normal distribution was obtained for control tissue (A) where for tumor tissue three normal distribution was found (B). The higher curve presented on the graph represents the sum of these three distributions (B).

The results of the study demonstrated that both genes are active in all analyzed tumors samples. Although the genes activity can be detected in control tissue lacking cancerous changes, the level of expression was significantly lower than the one found in cancer tissues (Figure 1 and 2). The differences between *CGB *and *GNRH1 *genes expression in cancer tissue and healthy tissue was found to be statistically significant (*P *= 0.000000 and *P = *0.001037, respectively).

**Figure 3:**
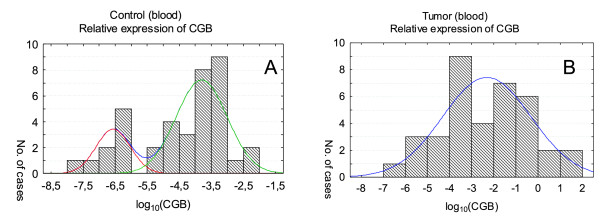
***CGB *expression in peripheral blood of healthy volunteers (A) and patients with cancer (B)**. Relative expression levels are presented as the logarithm to the base 10. *CGB *activity was fitted to two (A) and one normal distribution (B) in blood of healthy volunteers and cancer patients, respectively. The final approximation of *CGB *expression curve in control blood (A) is hidden due to the presence of non-overlapping components.

**Figure 4:**
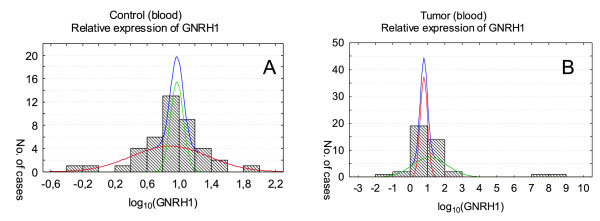
***GNRH1 *expression in peripheral blood of healthy volunteers (A) and patients with cancer (B)**. Relative expression levels are presented as the logarithm to the base 10. Analysis of *GNRH1 *expression blood of healthy volunteers (A) and patients with cancer (B) in both cases showed two distributions of results. The higher curve represents the sum of these two distributions.

*CGB *and *GNRH1 *transcripts were found also in peripheral blood of gynecological cancer patients as well as in blood of healthy volunteers (Figure 3 and 4). Nonetheless *CGB *expression in blood of healthy volunteers and patients with cancer differed significantly (*P = *0.001066) and was higher in blood of cancer patients. In case of *GNRH1 *analysis the difference of the gene activity between studied groups was not statistically significant; *P = *0.6098.

Due to the nature of the measurement real time RT-PCR data was log-transformed and then analyzed against existence of potential subpopulations varying in gene expression. Models of one, two and three coexisting subpopulations were taken into account and then evaluated using the maximal likelihood method. The outcome of this analysis was tested with F-test to assess the improvement of quality of the fit. Model of higher complicity (with greater number of subpopulations) was selected only if statistical significance of improvement (*P *< 0.05) was achieved. Additional verification of correctness of the chosen model was performed using Kolmogorov-Smirnov test. In this test all cases obtained *P *> 0.7. The final results showed that the model, which assumes the presence of more than one normal distribution components, is significantly better for describing heterogeneous expression of *CGB *and *GNRH1 *genes within studied groups.

In case of *CGB *expression analysis in tissues lacking cancerous changes only one distribution of results for each group was established (Figure 1A; Table 2). *CGB *expression in tumor tissues was categorized into two normal distributions (Figure 1B; Table 2). One of these distributions characterized by low level *CGB *activity (mean of log_10 _of *CGB *expression: -2.13, Table 2) corresponded to the results obtained for tumor blood (mean of log_10 _of *CGB *expression: -2.34, Table 2). The other one with distinctly higher level of the gene expression (mean of log_10 _of *CGB *expression: -1.35, Table 2) was typical for cancer tissue only.

**Table 2:** The distributions of *CGB *and *GNRH1 *genes expression within studied groups.

Material	I			II			III		
	
	Subpopulation [%]	Mean	SD	Subpopulation [%]	Mean	SD	Subpopulation [%]	Mean	SD
CGB Tumor (tissue)	36.8	-2.13	1.87	63.2	-1.35	0.62			

CGB Control (tissue)	100	-4.25	0.51						

CGB Tumor (blood)	100	-2.34	1.98						

CGB Control (blood)	24.2	-6.56	0.53	75.8	-3.80	0.79			

GNRH1 Tumor (tissue)	43.5	0.54	0.22	43.4	1.37	1.08	13.1	9.79	1.10

GNRH1 Control (tissue)	100	0.21	0.50						

GNRH1 Tumor (blood)*	49.0	0.79	0.21	46.0	1.13	1.00	5.0*	*	*

GNRH1 Control (blood)	63.0	0.88	0.48	37.0	0.97	0.08			

SD - standard deviation; * - two cases were excluded because they were found more then 10000 times higher than rightmost case from others.

The blood of cancer patients was characterized by one distribution of *CGB *expression only (Figure 3B) while blood of healthy volunteers was categorized into two subpopulations (Figure 3A).

*CGB *expression analysis in healthy volunteers' blood showed that this group can be divided into two subpopulations: one with low expression (smaller than -6.56) and the second one with high expression level of CGB (-3.80). The second population partially overlaps with distribution of *CGB *expression found for blood of cancer patients. Thus, in this particular case instead of usually using three sigma rules we applied -2.5 value to estimate the confidence limit, in which 95% of healthy volunteer had expression lower then critical value typical for cancer patients.

The raw results of *GNRH1 *expression were fitted to one, two or three coexisting subpopulations, each with normal distribution, and the model showed that one and two subpopulations can be set in control tissue lacking cancerous changes (Figure 2A) and control blood of healthy volunteers (Figure 4A), respectively (Table 2). In tumor tissue and blood of cancer patients three subpopulations with different levels of *GNRH1 *expression were established (Figure 2B and 4B).

Log-transformed results of *GNRH1 *expression in blood of cancer patient and in tumor tissue showed remarkably similar distributions (Figure 2B and 4B, Table 2). Two of these distributions found in tumor blood corresponded to lower level of the gene activity (*GNRH1 *mean in tumor blood: 0.79 and 1.13 and in tumor tissue: 0.54 and 1.37). Furthermore in both cases the distribution matched to extremely high activity of *GHNRH *(Figure 2B and 4B) was found.

For *GNRH1 *critical value was not established since the results of the gene expression in blood of cancer patients and healthy volunteers were overlapping.

No correlation between *CGB *and *GNRH1 *expression (Table 3) as well as clinical data (Table 4) in studied tissues and blood was observed.

**Table 3:** The correlation between *CGB *and *GNRH1 *genes expression within studied groups.

Material	CGB/GNRH1 *P *value
Tumor (tissue)	0.128

Control (tissue)	0.164

Tumor (blood)	0.115

Control (blood)	-0.089

Statistical significance *P *< 0.05.

**Table 4:** The correlation between *CGB *and *GNRH1 *genes expression in different cancer types.

Material	CGB/GNRH1 *P *value
Enodometrial cancer	0.961

Ovarian	0,234

Uterix	0,932

Statistical significance *P *< 0.05.

## Discussion

The critical role of circulating tumor cells in metastatic spread of carcinomas has already been very well documented. However the biology of these cells is poorly understood and the clinical relevance of their detection is still the subject of controversies. Available markers fail to distinguish between subgroups of CTC, and several current methods of CTC characterization and detection lack sensitivity, specificity and reproducibility [39].

Still early detection of these cells can become a useful method allowing the identification of cells with metastatic potential, and thus may be important for treatment and monitoring of cancer patients. RT-PCR based techniques and expression analysis of epithelial- and tissue-specific markers are the most sensitive methods for CTC detection. Results of numerous studies indicate that detection of single mRNA markers like mamoglobin, survivin, HER2, EGFR, VEGF and VEGFR range from 30 to 63% cases in peripheral blood of breast cancers. After combination of a few markers as one single panel the sensitivity usually increases [40]. A panel of six genes: *CCNE2*, *DKFZp1312*, *PPIC*, *EMP2*, *MAL2 *and *SLC6A8 *may serve as potential markers for CTC derived from breast, endometrial, cervical, and ovarian cancers [41]. Also mamoglobin gene expression is a sensitive molecular marker for tumor spread detection in not only in patients with breast cancer but also gynecological neoplasms [42]. CTC presence analyzed with Adna Breast Test (detection of EpCAM-, MUC-1-, and HER-2-transcripts) together with CA 125 assessment were shown to be of prognostic significance in gynecological cancers [43]. Similarly endothelial progenitor cell expressing CD43 and VEGFR2 circulating in the blood of patients with ovarian cancer may be a potential marker to monitor cancer progression and angiogenesis as well as treatment response [44].

Our study identifies two mRNA markers of gynecological cancers: human chorionic gonadotropin beta subunit (CGB) and gonadotropin releasing-hormone type 1 (GNRH1), which enable detection of circulating tumor cells.

We have previously demonstrated that CGB is a valuable marker of tumor tissue of uterine cervix, endometrium and ovary. *CGB *gene activity in cancer and atypical hyperplasia of endometrium is accompanied by the expression of gonadoliberin type 1, which physiologically stimulates the synthesis and secretion of gonadotropins [33-35].

In this study the presence of cells expressing *CGB *and *GNRH1 *in tumor tissue and blood of gynecological cancer patients was confirmed with real time RT-PCR. The results demonstrated that both genes are active in all analyzed tumor samples. *CGB *and *GNRH1 *transcripts were detected also in control tissue lacking cancerous changes, however the expression level of *CGB *gene in control group was significantly statistically lower than in cancer group. Similarly both genes expression was demonstrated in peripheral blood of gynecological cancer patients as well as in control group consisting of healthy volunteers' blood. The level of *CGB *expression in blood of cancer patients and in blood of healthy volunteers differed significantly while *GNRH1 *activity in the studied groups was not statistically significant.

Due to the nature of real time RT-PCR measurement the levels of *CGB *and *GNRH1 *relative expression were log-transformed and fitted to multiplied normal distribution model using the maximal likelihood method. The results of the conversions showed that the model assuming the presence of more than one normal distribution components improved the description of heterogeneous expression of studied genes.

Analysis of *CGB *and *GNRH1 *expression in tissue lacking cancerous changes showed one distribution of results for both genes. In case of tumor tissue *CGB *and *GNRH1 *activity were fitted into two and three normal distribution, respectively. The first population showing lower expression of *CGB *(mean of log_10 _of *CGB *expression: -2.13) consisted of 36.8% of tissues, while the second with higher *CGB *activity (mean of log_10 _of *CGB *expression: -1.35) included 63.2% of samples. Two distribution of *GNRH1 *with lower (mean: 0.54) and higher expression level (mean: 1.37) comprised of almost the same number of analyzed tissues (43.5%). The third distribution corresponded to the maximum gene activity with mean of log_10 _*GNRH1 *expression equal to 9.79 and includes 13% of examined samples. These samples may represent tissues producing maximal level of *GNRH1 *or tissue fragments containing higher number of cancer cells. Immunohistochemical analysis could verify these hypotheses

*CGB *and *GNRH1 *activity was studied also in blood of gynecological cancer patients and was compared to the control blood of healthy volunteers.

In control blood both genes were fitted into two distributions. However, *GNRH1 *distributions overlapped (mean: 0.88 and 0.97) and *CGB *distributions were separated from each other (mean: -6.56 and -3.8). The results showed that in case of *CGB *analysis in 95% of the population the gene expression is lower than -2.5, which indicates the lack of circulating tumor cells. In contrast 5% of control blood was shown to have *CGB *expression higher than -2.5. Thus, this critical value may be used to indicate the metastatic spread of tumor.

There is no defined explanation of *CGB *and *GNRH1 *activity noted both in control tissue lacking cancerous changes and blood of healthy volunteers. False-positive CG cases have been already reported before, though the elevated level of the hormone was detected only on protein level [45-48]. In these cases the presence of heterophilic antibodies was thought to be the reason for false-positive CG. In our study the activity of *CGB *and *GNRH1 *was detected on mRNA level. Sequence specific primers and hydrolysis probes used in real time PCR study excluded the possibility of false-positive results in case of both genes amplification. This implies that cells with altered gene expression can exist in healthy tissue. Even if the number of these cells is very small high sensitivity of real time RT-PCR enables their detection. Consequently, not only the presence of genes' transcripts but also the level of their expression should be verified in case of tumor cells detection.

Analysis of *CGB *expression transformed results in blood of gynecological patients revealed the presence of one distribution. One of the two distributions found in control group overlapped partially with *CGB *detected in cancer patients. Nonetheless maximal *CGB *expression level found is some cancer patients was 10^5 ^higher than maximal activity of the gene of given healthy volunteers. Thus, it may be concluded that the high activity of human chorionic gonadotropin beta subunits indicated the presence of tumor cells circulating in blood of patients.

The raw results of *GNRH1 *expression in blood of cancer patients was fitted to three normal distributions. Two of these distributions corresponding to lower level of the gene activity (mean of log_10 _of *GNRH1 *expression: 0.79 and 1.13) were similar to these observed in tumor tissue and control blood. Additionally in blood of cancer patients as well as in tumor tissue a third subpopulation corresponding to extremely high activity of *GNRH1 *(Figure 2B and 4B) was found. This activity was 10^5 ^higher than in other cases which may indicate patients in metastasis stage.

Analysis of results demonstrated that in part of the studied blood samples of cancer patients activity of *CGB *and *GNRH1 *was on the same level as in control group. There is no defined explanation of this fact, however some possibilities should be considered. The simplest one is based on the presumption that examined patients simply lacked CTC, which is probably especially that patients in early cancer stages were examined. Another possibility is that the cells were present but their number was so small that we were not able to detect them. In fact many authors admit to the inability to detect circulating tumor cells because of their small number, indicating insufficient capacity of CTC isolation methods [49]. Another possibility is that tumor progression enhances its heterogeneity, clonal selection, and variable expression of individual mRNA markers [50,51].

When designing this study, we assumed that cancer cells that spread from a primary tumor, and penetrate the bloodstream have metastatic potential and show a similar profile of gene expression to the cells present in the initial tumor mass. According to the theory of tumor cellular heterogeneity and its genetic instability once CTC detach from a primary tumor they may change their expression profile, adapting to new microenvironment [52]. What is more it can not be excluded that analysed gynecological cancer types might not metastasize primarily *via *the hematogenous route, thus CTC could be even rarer events than expected.

Still based on the results of analyzed genes activity in blood of volunteers and cancer patients the presence of cancer cells can be distinguished. High expression level in case of *CGB *and *GNRH1 *expression allowed identifying four and two individuals, respectively as cancer patients having tumor cell circulating in the blood flow. High *CGB *activity was found in blood of three patients with ovarian carcinoma (FIGO II, n = 1; III, n = 2) and one patient with endometrial cancer. *GNRH1 *expression was detected in two patients with ovarian carcinoma (FIGO II, n = 1; III, n = 1). The expression level of the genes assessed in blood of these patients was 10^5 ^higher than the genes activity observed in control group.

Our study demonstrated that CTC-related markers' expression may be heterogeneous therefore establishing a critical level of genes expression may be useful in order to recognize the spread of cancer cells. Defining such a "cutoff value" may be applied not only for *CGB *and *GNRH1 *expression but also other genes used as CTC markers. Especially that most of previously published data are limited to showing the percentage of positive cancer patients without any presentation of the number of positive healthy controls [40].

No correlation between *CGB *and *GNRH1 *expression in studied tissues and bloods as well as clinical data was observed (*P *> 0.05). This suggests that analyzed genes' expression profiles are independent of one another as well as of cancer type. The studies on the mechanisms regulating these genes activity may help explain the observed phenomenon.

## Conclusions

The assessment of human chorionic gonadotropin beta subunit and gonadoliberin type 1 expression levels in blood of cancer patients may allow distinguishing patients with tumor cells circulating in their blood and indicate the metastatic spread of these cells.

## Competing interests

The authors declare that they have no competing interests.

## Authors' contributions

AM, AS, AJ participated in the study design, carried out the molecular genetic studies and performed data analysis. AJ has been involved in coordination of the study and *drafting the manuscript*. MWC, WW performed the statistical analysis and interpretation of data. ENM, EG, KA collected surgical tissue and blood samples, performed anatomicopathologic macroscopic and microscopic examinations and delivered clinical patients' data. All authors read and accepted the final manuscript.
